# The Treatment of Intermittent Fever

**Published:** 1895-11

**Authors:** Walter M. Flemming

**Affiliations:** 240 Fifth Avenue, New York City


### Progress in Medicine.

### THE TREATMENT OF INTERMITTENT FEVER.

Before the discovery of the tubercle bacillus, many a cough was allowed to continue without treatment, and the real difficulty not suspected until a sharp hemorrhage or a hectic fever revealed the situation. Only a few months ago, diphtheria patients walked the streets with only a simple sore throat, while cases of follicular tonsillitis were carefully treated as diphtheritic in character. Nearly all of us were taught that malaria was an earth-born poison which filled the air with "a fever-generating agent."

Now all this is changed. The tubercle bacilli can be detected in the earliest stages of phthisis. The Klebs-Loefler bacillus decides the presence of diphtheria; and the malarial germs of Laveran tell us we have an intermittent fever.

It is natural that we look for a remedy which will destroy these germs, or counteract their poisonous products. Indeed, it now appears that we have, unconsciously to be sure, been giving a perfect specific for the cause of the latter disease. Quinine is no longer administered in an empirical manner. We know precisely why we give it and what it does. Quinine exerts a deathly influence over the malarial germ, therefore it may be given with the satisfaction of knowing that it will invariably check the paroxysms of an intermittent fever. If its use is not followed by this cure, then it is certain that it never came in contact with the red corpuscles of the blood. Absorption was incomplete, or the quantity of the drug was insufficient. In the light of all this, it is certainly bordering on the ludicrous that the National Dispensatory should give a list of seventy-six remedies in the index under intermittent fever.

The patient has usually had a malarial paroxysm before seeking medical advice. As hepatic activity is necessary to obtain the best effect of the specific treatment, so, in cases of constipation at least, it is better to begin the treatment with the following prescription, which should be taken five or six hours prior to the quinine:

℞ Hydrarg. Chloridi Mite,
Sodæ Bicarb.................. aa gr. I

M. Divide into six powders.

Sig.: Take one powder every fifteen minutes, using all the powders.

This is far preferable to giving the calomel in one single dose and in larger quantity. However, it may be necessary, if the patient is insensitive to purgatives, to increase the quantity in the prescription by one-half. While this preparatory treatment is not necessary, yet it is certainly true that after its employment a less quantity of quinine is required, and the general condition of the patient is improved.

It has been stated that this treatment should be given five or six hours before the specific treatment. It should now be said that the latter treatment is best inaugurated at such a time that it will be exerting its fullest physiological action when the next paroxysm is due. This time can be quite accurately stated when we remember that a paroxysm often begins an hour earlier than the preceding one, and that about three hours are required for the quinine to be in its most active condition in the body.

To insure prompt and complete absorption, the quinine is best given in liquid form. The following is a favorite prescription:

℞ Quinia Sulph.................... gr. xv
Aquæ.............................. oz. j
Acid Sulph., dil. q. s. ft. sol.

M. et Sig.: Take at one dose in one-third glass of water.

With the above preparatory treatment, and with the quinine dissolved, this dose is equivalent to at least twenty grains given after the usual manner; while it is certain we should not trust to pills or capsules at such a time, unless we know positively that these are in a perfectly soluble condition.

To prevent the uncomfortable head symptoms which accompany full doses of quinine, and also to relieve the pain which is likely to be present at the same time of the expected paroxysm, the following prescription should be given four or five hours after the specific:

℞ Antikamnia Tablets (5 grs. each) No. xxiv (24).

Sig.: One tablet every two hours while pain necessitates.

While the above dose of quinine is sufficiently large for residents of most parts of the United States, yet in some of the Southern States, and in other sections where malaria abounds with unusual force, it may be necessary to give the quinine as high as twenty, forty, or even sixty grains. But in the great majority of cases the above single dose will be sufficient to prevent a second chill.

In order that there may be no question about the recurrence of an attack, and also in order to bring the system under the influence of a good tonic, the quinine should be continued for one or two weeks, in doses of five to ten grains a day. As the malarial germ has left its effects on the nervous system, and often to a marked degree, so a remedy is indicated which will put at rest the disturbed condition. The following will be found satisfactory in every way:

℞ Antikamnia and Quinine Tablets, 5 grs. each, No. xxiv.

Sig.: One tablet three times a day after meals.

This tablet contains 2½ grs. sulphate quinine, and 2½ grs. antikamnia, being the most desirable proportion.

If the physician be called while the patient is suffering from a paroxysm, and he is in doubt as to its nature, he has only to remember that any intermittent fever which resists the action of quinine is not necessarily of malarial origin. Even during the chill of a malarial attack, the temperature may rise to 102° or higher, while it is often true that when the chill has passed and the fever is on, the thermometer will show a lower degree of heat. Therefore, no better treatment can be given at the beginning of or during the chill than the following:

℞ Antikamnia Tablets, 5 grs. each, No. xxiv.

Sig.: Take two tablets immediately. Repeat dose in two hours, if pain necassitates.

The antikamnia will relieve the congestion of the abdominal and thoracic organs, and will materially alleviate the headache of the second stage especially. In fact, it practically robs the fever of its most distressing features.

When we consider that the cause of intermittent fever is so thoroughly understood, and that quinine is regarded as its specific, destroying said cause, how puerile are all attempts to bring forward new substitutes. Although pain may not be dependent upon any special living organism, yet it is certain that in antikamnia we have a most reliable specific.

In regard to the treatment of all forms of febrile maladies, periodic or continued, I have found the antikamnia tablets, with its various combinations of codeine, salol or quinine (as indicated in each individual case), the most reliable, prompt and satisfactory remedies in controlling these intractable disorders of any remedial agent known to me in a general active practice of over thirty years.

WALTER M. FLEMMING, M. D.,
240 Fifth Avenue, New York City.

